# Cocaine: A Rare Cause of Left-Sided Renal Infarction

**DOI:** 10.1177/2324709615574907

**Published:** 2015-03-16

**Authors:** Padam Hirachan, Ravindra Agarwal, Brent Wagner

**Affiliations:** 1 Agarwal Nephrology and Hypertension PC, Columbus, GA, USA; 2 University of Texas Health Science Center at San Antonio, TX, USA

**Keywords:** cocaine, renal infarction, flank pain

## Abstract

Cocaine abuse is commonly associated with myocardial ischemia, mesenteric ischemia, and cerebrovascular accidents. Renal infarction is an uncommon complication of cocaine abuse. Various mechanisms have been postulated for this cocaine-related injury. There are only 15 cases reported on cocaine-induced renal infarction. Among the cases with available data, very few cases had left kidney involvement. We report a case of a 65-year-old African American man with history of cocaine abuse who presented with left flank pain and had left renal infarction.

## Background

Cocaine abuse is an epidemic in the United States, and its toxicity has been commonly associated with myocardial ischemia, mesenteric ischemia, and cerebrovascular accidents.^1^ Moreover, it is known to have detrimental effects on both acute and chronic renal failure. Various mechanisms have been postulated for cocaine-related injury, including changes in renal hemodynamics, glomerular matrix proliferation, oxidative stress, and induction of renal atherogenesis.^2^ Renal infarction secondary to cocaine abuse has been rarely reported in the literature and various mechanisms of this insults have been postulated.

## Case Presentation

A 65-year-old African American male with past medical history of hypertension, dyslipidemia, and spinal stenosis presented to emergency department for evaluation of persistent nausea, vomiting, and left flank pain for 3 days. There was no gross hematuria or dysuria. He also complained of decreased urine output for a day. The patient had recently visited the emergency department a day prior to this visit with the same complaint. He was then evaluated for nephrolithiasis with noncontrast computed tomography (CT) scan of abdomen and pelvis. The CT scan was unremarkable for any intra-abdominal pathology, and the patient was discharged home with pain medications. His home medications included lisinopril, chlorthalidone, and acetaminophen-hydrocodone. He was a chronic smoker of tobacco with a half pack per day and also smoked cocaine with last intake a day prior to presentation.

On physical examination, the patient was a well-built African American male with blood pressure of 171/91 mm Hg, pulse rate of 81 per minute, temperature of 98.4°F, and oxygen saturation was 98% on room air. Head, neck, heart and lung examinations were unremarkable. The abdomen was soft, nontender with active bowel sounds. However, there was mild left costoverterbral angle tenderness. There was no pedal edema, and neurological examination was unremarkable.

**Figure 1.**
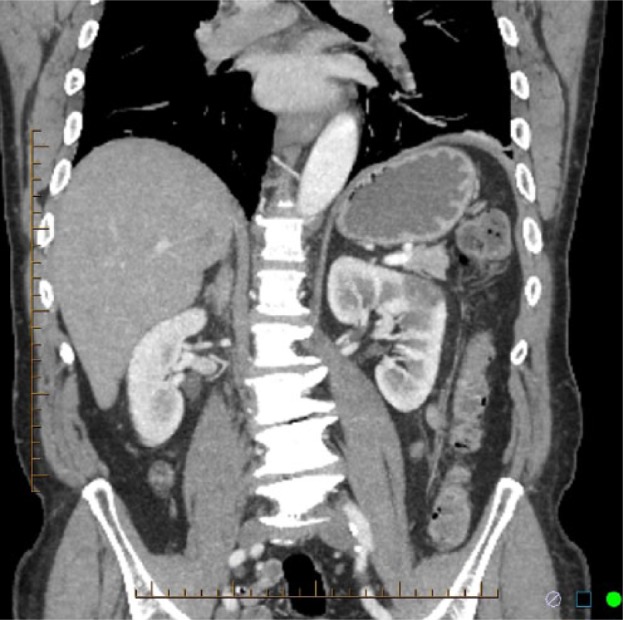
CT Scan of abdomen and pelvis showing wedge shaped non enhancing defect involving left inter polar region suggesting renal infarction.

**Figure 2.**
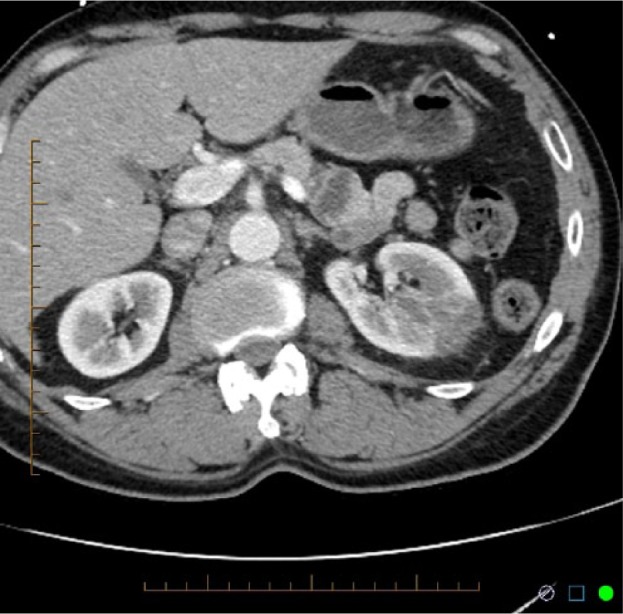
CT Scan of abdomen and pelvis showing wedge shaped non enhancing defect involving left inter polar region suggesting renal infarction.

Laboratory results on admission are shown in Table 1. Chest X-ray, electrocardiogram, and transthoracic echocardiogram were unremarkable. CT scan of abdomen and pelvis with intravenous contrast revealed a 4-cm, well-circumscribed, wedge-shaped nonenhancing defect involving the left inter polar region suggesting renal infarction (Figures 1 and 2). Renal ultrasound also showed localized edema within the mid left kidney suggesting subacute infarction, and color Doppler documented normal blood flow to both the kidneys. Further screening tests for hypercoagulability (factor V Leiden, prothrombin gene, protein C and protein S, antithrombin III, antiphospholipid antibody, and homocysteine); connective tissue disorder (anti–double stranded DNA, antinuclear antibody, serum complements including C3, C4); and lipid disorders were within normal limits (see Table 1).

**Table 1.** Serum and urine laboratory values at admission.

Laboratory Results	Values	Reference Range
White blood cell count	11.4 × 10^3^/µL	4.5-11 × 10^3^/µL
Hemoglobin	16.6 g/dL	13.5-17.5 g/dL
Blood urea nitrogen	30 mg/dL	6-20 mg/dL
Serum creatinine	1.6 mg/dL	0.9-1.5 mg/dL
Aspartate aminotransferase	63 IU/L	<42 IU/L
Alanine aminotransferase	27 IU/L	<40 IU/L
Albumin	4.6 g/dL	3.5-5 g/dL
Creatinine kinase	67 IU/L	<174 IU/L
Lactate dehydrogenase	1177 U/L	120-230 U/L
Urinalysis	Specific gravity 1.008; protein negative; blood negative	
Urine toxicology	Cocaine: reactive	

The patient was initially anticoagulated with heparin drip and later coumadin was started, until all the workups were available. The patient’s renal function subsequently improved and remained at baseline. The patient also underwent CT angiogram of abdomen/pelvis with evidence of patent renal artery and vein. Our patient was then diagnosed with cocaine-induced renal infarction, and his anticoagulation was eventually stopped.

## Discussion

Renal Infarction is an uncommon complication of cocaine abuse.^3,4^ Various mechanisms have been postulated in the literature.^1,2,5^ The most widely accepted hypotheses are cocaine-enhanced platelet aggregation, increased thromboxane synthesis, and endothelial and vasospastic injury due to inhibition of synaptosomal uptake of cathecolamines.^6^ Animal models have also shown that cocaine increases matrix accumulation, lowers intracellular gluthatione, and accelerates atherogenesis.^7-10^

Till date, there are only 15 cases reported on cocaine-induced renal infarction (Table 2). Among the cases with available data, there were 8 cases (7 males) with isolated involvement of right kidney, while 3 cases (males) had only left kidney involvement. Majority of the patients were middle-aged male.^5,11-13^ This gender predilection is likely secondary to high prevalence of cocaine use in males. It is presumed that right kidney is more prone to ischemia due to the increased resistance that it encounters by the longer length of its artery.^2^ Albeit rare, left kidney is no exception to this injury.

**Table 2.** Published Case Reports on Cocaine-Induced Renal Infarction.

Year of Publication (Reference)	Age (Years)	Gender	Kidney Involvement	Route of Cocaine Use
1984 (Sharif^14^)	32	Male	Right	Intravenous
1987 (Wohlman^15^)	32	Male	Right	Intravenous
1990 (Antonovych et al^16^)	39	Male	NA	NA
1993 (Kramer and Turner^17^)	37	Male	Right	Intravenous
1995 (Goodman and Rennie^18^)	37	Male	Right	Nasal
2001 (Saleem et al^19^)	25	Male	Right	Nasal
2003 (Mochizuki et al^20^)	52	Female	Right	Nasal
2004 (Edmondson et al^21^)	40	Male	Right	NA
2005 (Bemanian et al^2^)	48	Male	Right	Nasal
2007 (Caramelo et al^22^)	27	Male	Left	Intestinal transport
2008 (Furaz et al^23^)	36	Male	Bilateral	Nasal
2009 (Madhrira et al^5^)	47	Male	Bilateral	Nasal
2009 (Hoefsloot et al^11^)	36	Male	Left	NA
2011 (Le Guen et al^13^)	24	Male	Bilateral	Nasal
2012 (Fabbian et al^12^)	41	Male	Left	Nasal
Current report	65	Male	Left	Nasal

Abbreviation: NA, not available.

Renal infarction is a diagnostic challenge to the clinician due to its nonspecific clinical presentations and laboratory findings. Patients typically present with severe persistent flank and/or abdominal pain with or without nausea, vomiting, and fever. Typical laboratory findings include leucocytosis, microscopic hematuria, and elevated level of serum lactate dehydrogenase.^24,25^

Various imaging techniques including CT scan, ultrasound, magnetic resonant imaging, and nuclear scintigraphy scans have been used to make the diagnosis. However, contrast-enhanced CT scan is the noninvasive test of choice due to cost-effectiveness and widespread availability.^25^ There is no definitive treatment for acute renal infarction related to cocaine abuse. Prior treatment modalities in the literature included no treatment to anticoagulation, thrombolytic use, aspirin therapy, and surgical nephrectomy. Our patient was initially started on anticoagulation until all the hypercoagulable workups were reported negative. In conclusion, we report a rare case of left renal infarction secondary to cocaine abuse and presumably the fourth documented case report. Due to rare nature of the disease and nonspecific symptoms, a high degree of clinical suspicion is essential for early diagnosis of this rare condition.
